# Autoimmune/Inflammatory Syndrome Induced by Adjuvants With Sarcoid‐Like Pulmonary Lesions Triggered by Silicone Implant Rupture and Improved After Explantation

**DOI:** 10.1002/rcr2.70497

**Published:** 2026-02-02

**Authors:** Yuki Tsuda, Ryota Kikuchi, Nao Shioiri, Daigo Imasato, Waku Nakano, Taro Kufukihara, Mariko Kogami, Yuta Kono, Shinji Abe

**Affiliations:** ^1^ Department of Respiratory Medicine Tokyo Medical University Hospital Tokyo Japan

**Keywords:** adjuvants, ASIA, autoimmune, sarcoidosis, silicone implant

## Abstract

Silicone implants can trigger adjuvant‐induced autoimmune/inflammatory syndrome (ASIA). We present a 53‐year‐old woman who had undergone bilateral intramuscular silicone breast implantation at X−24 years. Following implant rupture at X year, she developed cough, fatigue, and arthralgia, accompanied by worsening sarcoid‐like pulmonary lesions. After implant removal, her clinical symptoms and radiological findings improved. Histopathological examination findings identified foreign‐body granulomas, fulfilling diagnostic criteria for ASIA. This case suggests that silicone leakage after implant rupture, rather than the duration of exposure, is a critical factor in disease development and progression. The sarcoid‐like pulmonary lesions in this case may represent a sarcoid‐like granulomatous reaction triggered by silicone exposure in the setting of ASIA, rather than true systemic sarcoidosis. Early implant removal appears beneficial for both the diagnosis and treatment of ASIA.

## Graphical abstract

Silicone implant rupture can trigger an autoimmune/inflammatory syndrome induced by adjuvants, presenting with sarcoid‐like pulmonary lesions. In this case report, removal of the ruptured implants led to clinical and radiological improvements.

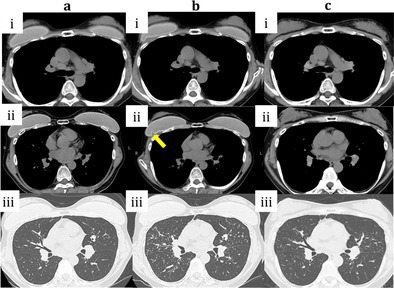

## 1 Introduction

Autoimmune/inflammatory syndrome induced by adjuvants (ASIA), first proposed by Shoenfeld et al. in 2011, is a systemic autoimmune and inflammatory reaction following exposure to adjuvants such as silicone implants, vaccines, or metals [1]. Its clinical manifestations are diverse and include arthralgia, chronic fatigue, neurological symptoms, and granulomatous lesions. In the respiratory field, an association between silicone exposure and sarcoid‐like pulmonary disease has been increasingly recognised [2]. We report a case of ASIA in which rupture of silicone implants led to the exacerbation of sarcoid‐like pulmonary lesions, which subsequently improved after implant removal.

## 2 Case Report

**FIGURE 1:**
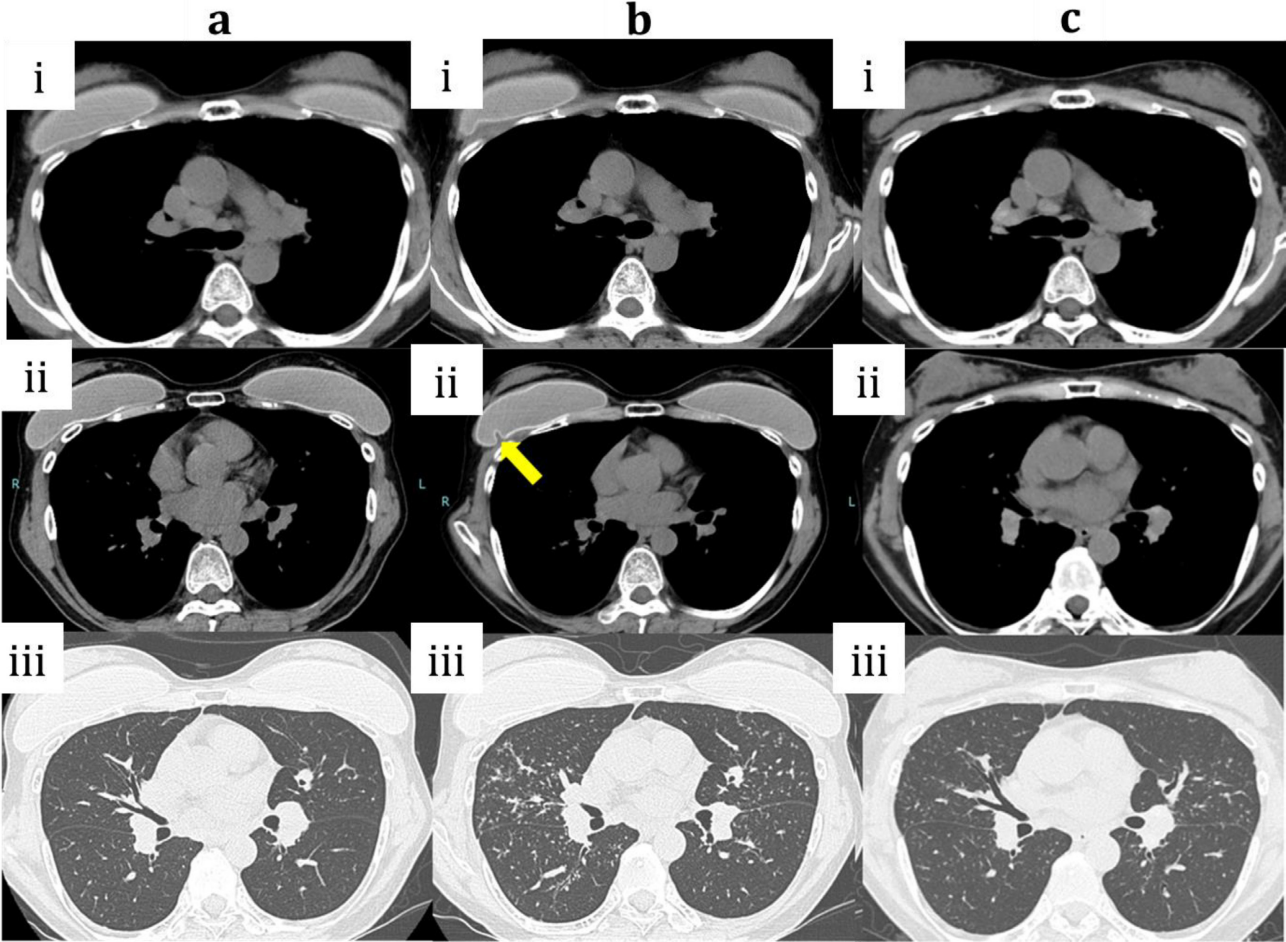
Chest CT findings. (a‐i) Mediastinal window: mediastinal and hilar lymphadenopathy is present. (a‐ii) bilateral subpectoral silicone implants are visible. (a‐iii) Lung window: subtle bilateral granular opacities are observed. (b‐i) Mediastinal window: mediastinal and hilar lymphadenopathy is slightly reduced in size compared with panel (a‐i), but remains enlarged. (b‐ii) deformation of the right silicone implant is evident (arrow). (b‐iii) Lung window: the granular opacities were distributed mainly along the bronchovascular bundles, consistent with a peribronchovascular pattern. (c‐i) Mediastinal window: mediastinal and hilar lymphadenopathy persists, with no significant change in size compared with panel (b‐i). (c‐ii) both implants have been removed. (c‐iii) Lung window: decreased density of bilateral granular opacities indicating improvement. CT, computed tomography.

**FIGURE 2:**
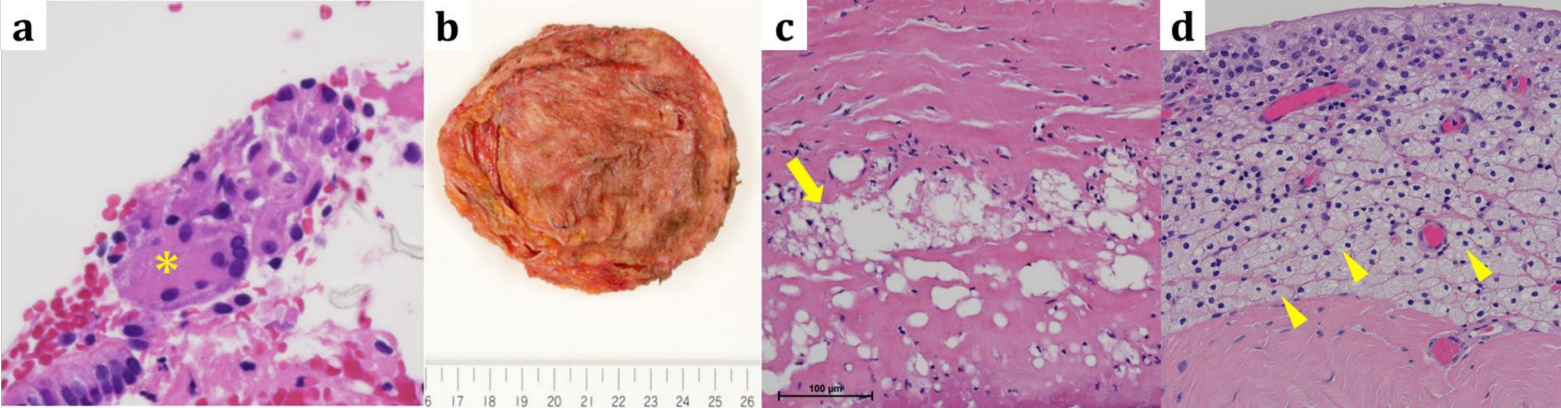
Histopathological findings. (a) Right lung tissue (high‐power field, ×600): multinucleated giant cells are observed (asterisk) (haematoxylin–eosin stain). (b) Gross appearance of the explanted right silicone implant. The outer shell is shown, with focal attachment of surrounding tissue including skeletal muscle fibres. (c) Peri‐implant tissue (high‐power field, ×400): amorphous silicone deposits (arrow) (haematoxylin–eosin stain). (d) Peri‐implant tissue (high‐power field, ×200): aggregates of foamy macrophages are observed (arrowhead), with only a very small number of lymphocytes and plasma cells. No neutrophils are identified. (haematoxylin–eosin stain).

A 53‐year‐old woman (after gender‐affirming surgery) with no history of systemic disease, medication use, smoking, or allergies underwent bilateral intramuscular silicone breast implantation at X−24 years. At X−4 years, she developed left uveitis. At X−3 years, chest computed tomography (CT) revealed mediastinal and hilar lymphadenopathy with subtle granular opacities (Figure 1a), and she was referred to our department for suspected sarcoidosis. Blood tests, including complete blood count, liver and renal function tests, electrolytes, and C‐reactive protein, were within normal limits. Laboratory test findings specific to sarcoidosis were as follows: angiotensin‐converting enzyme, 4.9 U/mL; soluble interleukin‐2 receptor, 372 U/mL; lysozyme, 4.9 μg/mL; and antinuclear antibodies were negative. Bronchoalveolar lavage fluid showed lymphocytosis (22%) with a CD4/CD8 ratio of 2.04, which was within normal limits. Transbronchial lung biopsy revealed noncaseating epithelioid granulomas (Figure 2a). The patient was followed up conservatively without any respiratory symptoms.

At X year, chest CT demonstrated deformation of the right implant (Figure 1b), coinciding with new‐onset cough, fatigue, and arthralgia. At that time, mediastinal and hilar lymphadenopathy showed a slight decrease in size compared with previous imaging, although lymph node enlargement was still present. Pulmonary granular opacities progressed, predominantly with a bilateral peribronchovascular distribution. Vital signs were stable, as follows: blood pressure, 130/93 mmHg; heart rate, 72 bpm; respiratory rate, 18/min; SpO_2_, 99% (room air); and temperature, 35.6°C. A bilateral implant removal was performed. Rupture of the outer shell was observed in the explanted right silicone implant (Figure 2b). Histopathological examination of the explanted capsule showed deposition of an amorphous, non‐stained material consistent with silicone, accompanied by clusters of foamy macrophages and multinucleated giant cells (Figure 2c), indicating foreign‐body granuloma formation secondary to implant rupture. Postoperatively, her symptoms improved rapidly and follow‐up CT showed marked resolution of the granular opacities (Figure 1c).

## 3 Discussion

According to Shoenfeld's criteria, ASIA can be diagnosed when two or more of the following are present: adjuvant exposure, typical clinical manifestations, improvement after removal, and histological evidence of foreign‐body granulomas [1]. This patient met all of the criteria and presented with cough, fatigue, and arthralgia after long‐term silicone exposure, improved following implant removal, and histological confirmation of foreign body granulomas.

In sarcoidosis, T helper type (Th)1‐dominant immune activation and macrophage‐mediated granulomatous inflammation play central roles in the pathogenesis. Similar immune mechanisms have been implicated in ASIA, in which chronic silicone‐induced inflammation may trigger Th1/Th17 responses and promote granuloma formation [2]. Epidemiological data suggest that patients with silicone implants have approximately twice the risk of developing sarcoidosis as the general population [3]. Both ASIA and sarcoidosis share a common immunopathological basis involving persistent immune activation by exogenous antigens or foreign materials. In line with this concept, the granulomas identified both around the implant and within the lung in our patient likely reflected shared immunological pathways, supporting a common pathophysiological mechanism. In our case, the BALF CD4/CD8 ratio was within normal limits (2.04), which is lower than the typically elevated values reported in classic pulmonary sarcoidosis. Taken together with the clinical course and the presence of peri‐implant foreign‐body granulomas, these findings are more consistent with a sarcoid‐like granulomatous reaction associated with silicone exposure in the setting of ASIA, rather than true systemic sarcoidosis.

In this case, the patient did not report any breast swelling or pain despite implant rupture. The immune response to silicone may therefore have manifested predominantly as a lymphatic and systemic reaction rather than as localised breast inflammation. Silicone particles can migrate via lymphatic channels to regional lymph nodes and induce a Th1‐dominant granulomatous immune response [4]. Accordingly, the absence of breast symptoms may reflect preferential lymphatic dissemination and immune processing of silicone material rather than overt local inflammation. Watad et al. reported that the most frequent ASIA symptoms were arthralgia (64.8%), chronic fatigue (54.5%), and myalgia (34.9%) [5], consistent with our patient's presentation. Ruptured implants are associated with a higher frequency of systemic symptoms than intact implants [6]. In the present case, the patient remained asymptomatic until the implant ruptured, after which systemic and pulmonary manifestations developed. The average latency period for ASIA is reported to be approximately 31 months [5]; however, in our case, it occurred > 20 years after implantation, suggesting that abrupt silicone leakage, rather than cumulative exposure, may serve as a key trigger for disease onset and exacerbation. Therefore, the early removal of ruptured implants can be both diagnostic and therapeutic. Moreover, improvement in clinical and radiological findings following explantation highlights the reversibility of immune activation after removal of the initiating adjuvant.

In this case, mediastinal and hilar lymphadenopathy with subtle lung granular opacities were already present 3 years before the clinical exacerbation, suggesting an early phase of immune sensitization related to chronic silicone exposure. These findings indicate that minor shell damage may have preceded the overt implant rupture, resulting in low‐level silicone leakage and gradual immune sensitization with sarcoid‐like changes. Following implant rupture, a sudden increase in silicone leakage may have triggered overt systemic symptoms and progression of pulmonary lesions.

Clinically, when encountering patients with unexplained pulmonary nodules or granulomatous lesions, physicians should consider ASIA as a differential diagnosis along with sarcoidosis. A thorough history of cosmetic procedures or foreign material implantation is essential because an adjuvant exposure history often provides diagnostic clues. With the increasing prevalence of cosmetic implant procedures, the recognition of ASIA is expected to increase. As ASIA is potentially reversible with adjuvant removal, early identification through a detailed exposure history is crucial.

In conclusion, here, we describe a case of ASIA presenting with sarcoid‐like pulmonary disease triggered by silicone implant rupture. The patient's clinical and radiological findings improved after implant removal, suggesting that early explantation is beneficial for both diagnosis and treatment.

## Author Contributions

Y.T. and R.K. contributed to the conception and drafting of the manuscript. N.S., D.I., W.N., T.K. and M.K. assisted in data collection and literature review. S.A. supervised the work and critically reviewed the manuscript. All authors approved the final version of the manuscript.

## Funding

The authors have nothing to report.

## Ethics Statement

This report was prepared in accordance with the ethical standards of our institution and the Declaration of Helsinki.

## Consent

The authors declare that written informed consent was obtained for the publication of this manuscript and accompanying images using the consent form provided by the Journal.

## Conflicts of Interest

The authors declare no conflicts of interest.

## Data Availability

The data that support the findings of this study are available on request from the corresponding author. The data are not publicly available due to privacy or ethical restrictions.
